# Sunitinib and Imatinib Display Differential Cardiotoxicity in Adult Rat Cardiac Fibroblasts That Involves a Role for Calcium/Calmodulin Dependent Protein Kinase II

**DOI:** 10.3389/fcvm.2020.630480

**Published:** 2021-02-01

**Authors:** Calum J. McMullen, Susan Chalmers, Rachel Wood, Margaret R. Cunningham, Susan Currie

**Affiliations:** Strathclyde Institute of Pharmacy and Biomedical Sciences, University of Strathclyde, Glasgow, United Kingdom

**Keywords:** sunitinib, cardiac fibroblast, CaMKII, cardiotoxicity, imatinib

## Abstract

**Background:** Tyrosine kinase inhibitors (TKIs) have dramatically improved cancer treatment but are known to cause cardiotoxicity. The pathophysiological consequences of TKI therapy are likely to manifest across different cell types of the heart, yet there is little understanding of the differential adverse cellular effects. Cardiac fibroblasts (CFs) play a pivotal role in the repair and remodeling of the heart following insult or injury, yet their involvement in anti-cancer drug induced cardiotoxicity has been largely overlooked. Here, we examine the direct effects of sunitinib malate and imatinib mesylate on adult rat CF viability, Ca^2+^ handling and mitochondrial function that may contribute to TKI-induced cardiotoxicity. In particular, we investigate whether Ca^2+^/calmodulin dependent protein kinase II (CaMKII), may be a mediator of TKI-induced effects.

**Methods:** CF viability in response to chronic treatment with both drugs was assessed using MTT assays and flow cytometry analysis. Calcium mobilization was assessed in CFs loaded with Fluo4-AM and CaMKII activation *via* oxidation was measured *via* quantitative immunoblotting. Effects of both drugs on mitochondrial function was determined by live mitochondrial imaging using MitoSOX red.

**Results:** Treatment of CFs with sunitinib (0.1–10 μM) resulted in concentration-dependent alterations in CF phenotype, with progressively significant cell loss at higher concentrations. Flow cytometry analysis and MTT assays revealed increased cell apoptosis and necrosis with increasing concentrations of sunitinib. In contrast, equivalent concentrations of imatinib resulted in no significant change in cell viability. Both sunitinib and imatinib pre-treatment increased Angiotensin II-induced intracellular Ca^2+^ mobilization, with only sunitinib resulting in a significant effect and also causing increased CaMKII activation *via* oxidation. Live cell mitochondrial imaging using MitoSOX red revealed that both sunitinib and imatinib increased mitochondrial superoxide production in a concentration-dependent manner. This effect in response to both drugs was suppressed in the presence of the CaMKII inhibitor KN-93.

**Conclusions:** Sunitinib and imatinib showed differential effects on CFs, with sunitinib causing marked changes in cell viability at concentrations where imatinib had no effect. Sunitinib caused a significant increase in Angiotensin II-induced intracellular Ca^2+^ mobilization and both TKIs caused increased mitochondrial superoxide production. Targeted CaMKII inhibition reversed the TKI-induced mitochondrial damage. These findings highlight a new role for CaMKII in TKI-induced cardiotoxicity, particularly at the level of the mitochondria, and confirm differential off-target toxicity in CFs, consistent with the differential selectivity of sunitinib and imatinib.

## Introduction

Cardiotoxicity is a recognized adverse effect of many clinically important drugs and has led to the withdrawal of a number of agents after their introduction to the market (1). Anti-cancer drugs are prevalent among the drug categories that exhibit cardiotoxicity and this can seriously impact upon cancer patient survival. In the long term, patients receiving anti-cancer drugs may be at a greater risk of death from cardiovascular disease than cancer (2). Conventional approaches for assessing cardiotoxic effects are similar to the indices used for assessing heart disease, including measurement of cardiac troponin and natriuretic peptides (3, 4). These markers usually only show significant changes after damage to the heart has occurred. Consequently, there is a real need to identify early onset biomarkers and signaling pathways that are switched on soon after initiating anti-cancer therapy and that correlate with adverse effects on the heart. This will be essential for the development of safer anti-cancer drugs in the future.

A prominent and well-recognized feature of anti-cancer drug-induced cardiotoxicity in cardiac myocytes (CMs) is the accumulation of damaging reactive oxygen species (ROS). Functional effects of ROS accumulation include CM hypertrophy, impaired contraction, apoptosis and autophagy, all of which are key features of cardiotoxicity (5). Importantly though, drug-induced cardiotoxicity does not only affect the contractile cells of the heart. Evidence is emerging that anti-cancer drugs can also have off-target damaging effects on other cells of the cardiovascular system including cardiac fibroblasts (CFs), endothelial cells (ECs), vascular smooth muscle cells (VSMCs), immune cells and cardiac progenitor cells (6). In a healthy heart there is considerable functional interaction between different cell types, particularly between the CMs and CFs. The latter cell type not only secrete chemical mediators that can influence cardiac contractility, but also provide components of extracellular matrix that ensure structural stability (7). In cardiovascular disease, CFs play a pivotal role in cardiac remodeling and in the accompanying contractile dysfunction by exhibiting hyper-proliferation and producing excess collagen. The resulting fibrosis is a key contributor to cardiac dysfunction (8). Recent studies have suggested that anti-cancer therapies can mirror the effects observed in CFs from diseased hearts, including increased oxidative stress and ROS accumulation similar to that seen in CMs but resulting in cardiac fibrosis and potential diastolic dysfunction (9–11).

Among the more novel anti-cancer agents to emerge in recent years are those that prevent cancer cell proliferation and angiogenesis *via* “selective” targeted tyrosine kinase and vascular endothelial growth factor receptor (VEGFR) inhibition. Unfortunately, these targeted therapies cause cardiotoxicity *via* both on-target (blocking VEGFR in cardiac cells) and off-target (non-VEGFR) effects (12). Understanding the cellular mechanisms that underpin these cardiotoxic effects will be crucial in improving the safety profile of tyrosine kinase inhibitors (TKIs), especially since these drugs are currently the standard treatment for several types of malignancy. The extent of TKI-induced cardiotoxicity may vary considerably across different members of the TKI drug group. Understanding the fundamental reasons for these differences is essential for tailoring future safer treatments. It is possible that different cardiotoxic responses may reflect an individual drug's capacity for on-target vs. off-target effects (13).

Two well-known TKIs that are currently in clinical use are imatinib and sunitinib. Imatinib mesylate is used for the treatment of chronic myeloid leukemia, gastrointestinal stromal tumors and hypereosinophilic syndrome. Imatinib works by targeting the ABL ATP binding site, stabilizing the inactive form of ABL, preventing tyrosine autophosphorylation and therefore kinase phosphorylation of substrates in cancerous cells, ultimately inducing apoptosis (14). There have been various reports of cardiotoxicity associated with imatinib. At a cellular level the cardiotoxic response has been suggested to include apoptosis as well as necrosis of CMs and this is as a result of mitochondrial dysfunction, ATP depletion and cytochrome C release (15). However, there is speculation over whether the concentrations of imatinib that exert the toxic effects reported on cardiac cells are clinically relevant (16). Despite evidence for cardiotoxicity, overall adverse effects on the heart following imatinib treatment appear relatively uncommon, ranging from 0.5 to 1.7% (17, 18). Sunitinib malate (SUTENT®) on the other hand has been reported to exert a wide range of cardiotoxic effects. Reports of hypertension and congestive heart failure in patients receiving sunitinib therapy ranges from 17 to 43% and 3 to 18% (19) respectively. Sunitinib is commonly used to treat renal cancers and has shown considerable benefit in patients with metastatic renal carcinoma (20). Sunitinib has been suggested to have a number of off-target effects that can adversely affect the heart. Examples of off-target effects include inhibition of AMP-activated protein kinase (21) and activation of calcium/calmodulin dependent protein kinase (CaMKII) (22), both of which, if chronically affected, can result in cardiac dysfunction.

Previous work by our group has highlighted that CaMKII activation occurs in guinea pig hearts following chronic administration of clinically relevant concentrations of either imatinib or sunitinib (22). Increased cardiac CaMKIIδ expression and CaMKII activity were shown to occur in the absence of any overt cardiac dysfunction. We suggested that these changes could reflect early adaptations by the heart to these TKIs which could precede the onset of contractile dysfunction. As such, we suggested that CaMKII might be a useful early onset marker for TKI-induced cardiotoxicity. Our original work comprised *in vivo* assessment of cardiac function following acute and chronic TKI treatment. CaMKII was assessed in whole cardiac homogenate preparations from TKI-treated hearts. Crucially, there was no investigation of specific cellular responses to either imatinib or sunitinib, evidence that we need to inform us upon more tailored clinical treatments going forward. In order to characterize the cardiotoxic effects of imatinib and sunitinib in more detail, the current study has, for the first time, compared the effects of both TKIs on adult rat CFs across a range of clinically relevant concentrations. Cell phenotype and viability have been assessed along with effects on mitochondrial superoxide production. Here we present novel evidence for distinct differences in the cellular responses to both drugs in CFs. Importantly, we also identify a common role for CaMKII in both imatinib and sunitinib-mediated mitochondrial superoxide production.

## Methods

### Cardiac Fibroblast Isolation

All procedures were performed under sterile conditions and conformed to local ethical review committee guidelines as well as to the Guide for the Care and Use of Laboratory Animals published by the US National Institutes of Health (NIH Publication No. 85-23, revised 1996) and Directive 2010/63/EU of the European Parliament. CFs were isolated from male Sprague-Dawley rats weighing between 250 and 350 g. Rats were sacrificed *via* cervical dislocation and ventricular CFs isolated under sterile conditions *via* the bulk collagenase digestion method described previously (23). Briefly, isolated hearts were washed in Ca^2+^ free Krebs solution (120 mM NaCl, 5.4 mM KCl, 0.52 mM NaH_2_PO_4_, 20 mM HEPES, 11.1 mM glucose, 3.5 mM MgCl_2_, 20 mM taurine, 10 mM creatine) supplemented with 1 mM EGTA, 1% (v/v) penicillin/streptomycin; pH 7.4. The aorta and atria were then excised before the remaining ventricular tissue was finely chopped in a digestion buffer containing 0.8 mg/ml collagenase type 1 (Worthington Chemical, UK) and 0.3 mg/ml protease XIV (Sigma-Aldrich) prepared in Ca^2+^ free Krebs solution. The isolated CFs were resuspended in CF growth medium [DMEM supplemented with 20% (v/v) FBS, 2% (v/v) penicillin/ streptomycin and 1% (v/v) L-glutamine] before being plated in a T75 culture flask and incubated (37°C, 5% CO_2_). The CF growth media was replenished after 4–5 h of incubation to remove non-adherent cells. The media was replenished 24 h post-isolation to remove any remaining cell debris. Subsequent media changes conducted at 48 h intervals thereafter as per the standard cell culture protocol until 70–80% confluent.

### Drug Treatment

CFs were grown to ~75% confluence in CF growth medium (outlined above) and were treated with sunitinib malate and imatinib mesylate at the indicated concentrations for 18 h in the same CF growth medium containing 20% serum (outlined above). Stock concentrations of drugs were diluted in DMEM supplemented with 2% (v/v) penicillin/streptomycin and 1% (v/v) L-glutamine prior to addition. Working concentrations were based on clinically relevant concentrations previously published (22, 24). Where appropriate, 5 μM KN-93 was added for 2 h pre-treatment prior to the addition of drug for 18 h.

### Imaging of Cells

Cell phenotype and growth was monitored using a Nikon Eclipse (TE300) inverted microscope (Nikon, Tokyo, Japan) and a Leica EC3 digital camera affixed to a Leica DM IL LED inverted microscope at 10 × magnification (Leica Biosystems, Wetzlar, Germany).

### Immunofluorescence

CFs, human umbilical vein endothelial cells (HUVECs), colonic smooth muscle cells (SMCs) and myofibroblasts (MFs) were seeded in 12-well plates containing 13 mm glass coverslips at a density of 1.5 × 10^4^ cells/ml and cultured until 40–60% confluent. MFs were obtained following passage of CFs to p4 or p5 and were initially identified visually based on dramatic change in cell phenotype where MFs were significantly larger and rounder. Cells were fixed in 3.6% (v/v) formaldehyde in phosphate buffered saline (PBS) for 10 min at room temperature before being permeabilised with 0.25% Triton X-100 in PBS for 10 min at room temperature. Cells were then blocked in 1% (w/v) BSA in PBS for 30 min before incubation with primary antibody diluted in 1% (w/v) BSA in PBS overnight at room temperature [vimentin 1:400; Sigma-Aldrich (V5255)], von Willebrand factor [vWF; 1:100; Abcam (ab6994)] and smooth muscle cell actin [SMCa; 1:200; Abcam (ab7817)]. Following overnight incubation, coverslips were washed three times with PBS before being blocked in 1% (w/v) BSA in PBS for 15 min at room temperature. Coverslips were then incubated (covered) with the corresponding Alexa Fluor™ 488 secondary antibody {1:100; Thermo Fisher, Renfrew, UK [Mouse (A-11001); Rabbit (A-11008)]} in 1% (w/v) BSA in PBS for 1 h at room temperature. Following further washes in PBS, samples were stained with DAPI (1:2,000) for 5 min before being mounted onto glass coverslips with Mowiol® and stored at 4°C until imaged. Samples were imaged using the EVOS™ FL Auto Imaging System (Thermo Fisher) at 20 × magnification.

### MTT Assays for Cell Death Determination

CFs were seeded in a 96-well plate at a density of 2.5 × 10^4^ cells/well and cultured in growth medium for 24 h prior to drug treatment. Treated samples were incubated with 3-(4,5-dimethylthiazol-2-yl)-2, 5-diphenyltetrazolium bromide MTT (10 μg/ml) prepared in fresh growth medium for 2 h (37°C, 5% CO_2_). In living cells, mitochondrial dehydrogenases convert MTT to purple MTT formazan crystals. The purple formazan crystals were resuspended in DMSO and cell viability assessed *via* optical density at a wavelength of 570 nm. Data was expressed as a percentage of control untreated cells.

### Flow Cytometry (FC) Analysis

CFs were seeded in a 12-well plate at a density of 5 × 10^4^ cells/well and cultured in growth medium until 70% confluent and then treated as per the *in vitro* drug treatment protocol. CFs were also treated with H_2_O_2_ for 18 h to provide single stain controls (600 μM H_2_O_2_ annexin V (AnV) control and 1.5 mM H_2_O_2_ propidium iodide (PI) control). Once treated, the media from each well was collected into individual FACS tubes. Samples were then dissociated using TrypLE™ Express (37°C) and collected into their respective FACS tubes alongside the previously collected media. Wells were then washed with PBS and added to the corresponding FACS tube to ensure all cells were recovered before being centrifuged at 1,000 g for 5 min. The samples were washed with PBS and 100 μl of AnV binding buffer (BD Biosciences, UK) added to each FACS tube. AnV (BD Biosciences, UK) (5 μl) was then added to all FACS tubes except the control (unstained) and the PI single stain (1.5 mM H_2_O_2_ treated) samples. The samples were then covered with aluminum foil to omit light and incubated for 15 min at room temperature. Following incubation, 400 μl of 1:500 PI [Thermo Fisher (P3566)] in AnV binding buffer was added to all FACS tubes except the control (unstained) and annexin V single stain (600μM H_2_O_2_ treated) samples. 400μl of AnV binding buffer was added to the control (unstained) and AnV single stain (750μM H_2_O_2_ treated) samples. The samples were then analyzed using the BD FACSCanto flow cytometer (BD Biosciences, California, USA) and FlowJo v9 (FlowJo LLC, Oregon, USA).

### Western Blotting

For assessing Total-CaMKIIδ and Phos-CaMKII expression, CFs were lysed in 150 μl of LDS sample buffer [25% (v/v) 4 × Nu PAGE LDS Sample Buffer (Thermo) and 75 mM DTT] and denatured at 100°C for 5 min. For assessing Ox-CaMKII expression, CFs were lysed in the absence of the reducing agent DTT. Protein lysates were loaded onto 4–20% Mini-PROTEAN TGX Precast Protein Gels and subjected to electrophoresis at 120 V for 110 min in running buffer (3.5 mM SDS, 192 mM glycine, and 25 mM tris base) using a Mini-PROTEAN Tetra chamber (Bio-Rad Laboratories Ltd., UK) at room temperature. Proteins were then transferred to nitrocellulose membranes (0.45 μm pore, GE Healthcare, UK) in transfer buffer [192 mM glycine, 25 mM tris base, 20% (v/v) methanol] using a MiniTrans-Blot Electrophoretic Transfer Cell (Bio-Rad Laboratories Ltd., UK) at a constant current of 280 mA for 110 min at room temperature. Membranes were then blocked in 5% (w/v) BSA in TBS-T [20 mM Tris base, 150 mM NaCl, adjusted to pH = 7.4, and 0.1% (v/v) Tween 20] for 120 min at room temperature. The membranes were then incubated with the appropriate primary antibody in 0.5% (w/v) BSA in TBS-T (0.1%) overnight at 4°C {Ox-CaMKII [1:5,000; Insight biotechnology Limited (GTX36254)]; Phos-CaMKII [1:5,000; Badrilla (A010-50)]; CaMKIIδ [1:5,000; Eurogentec (custom made)]; GAPDH [1;100,000; Abcam (ab8245)]}. Membranes were then washed in TBS-T (0.1% Tween) followed by incubation in the corresponding secondary antibody (1,5,000; Jackson ImmunoResearch, Cambridge, UK [Mouse (715-035-150); Rabbit (111-035-144)] in 0.5% (w/v) BSA in TBS-T (0.1% Tween) for 90 min at room temperature. This was followed by a further wash in TBS-T (0.1% Tween) before being developed *via* enhanced chemiluminescence and exposure onto X-ray film. For re-probing purposes, membranes were stripped in Tris-based buffer (31 mM Tris base, 35 mM SDS containing 0.7%, pH = 6.7). X-ray film was then scanned on a HP Deskjet 2540 printer scanner and densitometry was carried out using Image J software (25). Blotting data was normalized as indicated in the relevant figure legends and samples processed as percentage of control untreated signal.

### Intracellular Calcium Release

Cells were seeded into black sided, clear bottomed 96-well plate at a density of 2.5 × 10^4^ cells/well and cultured for 24 h before treatment with sunitinib and imatinib as described previously. Culture medium was aspirated and the cells washed twice with HBSS (136.9 mM NaCl, 5.4 mM KCl, 1.3 mM CaCl_2_, 0.4 mM MgSO_4_·7H_2_O, 0.5 mM MgCl_2_.6H_2_O, 0.3 mM Na_2_HPO_4_.2H_2_O, 0.4 mM KH_2_PO_4_, 5.6 mM glucose, 4.2 mM NaHCO_3_). Cells were then incubated with 2 μM Fluo4-AM Ca^2+^ indicator dye in Fluo4 buffer [1 mM MgCl, 1.5 mM CaCl, 0.3 mM probenecid, and 0.1% (w/v) BSA in HBSS] for 2 h (37°C, 5% CO_2_). Cells were then washed twice with Fluo4 buffer before adding 80 μl of Fluo4 buffer to each well. A compound plate was prepared using the ligands of interest, diluted to 3 × the final concentration in Fluo4 buffer. The plate reader transfers 40 μl of compound to each well, diluting the compound 1:3 to reach a 1 × desired final concentration for each sample. Fluorescence excitation was then measured using a Flexstation 3 microplate reader (Molecular Devices, Wokingham, UK) at Ex:494/Em:525 nm and SoftMax® Pro software, version 5.4.3.

### MitoSOX Live Cell Imaging

MitoSOX fluorescence was recorded on a Nikon TiE inverted epifluorescence microscope with 40 × 1.3 NA oil immersion objective, illuminated with 515 nm excitation light (Pe4000 multi-LED; CoolLED, Andover, UK) and emitted light >550 nm selected by dichroic mirror and captured on an ORCA Flash 4.0 CMOS camera (Hamamatsu, Welwyn Garden City, UK). Cell imaging chambers were placed inside a stage-top incubator (H301; OKO labs, Naples, Italy) and maintained at 37°C throughout the imaging process. CFs were seeded into the 8-well chambers at a density of 2.5 × 10^4^ cells/well, cultured in CF growth medium for 24 h and then treated as per the *in vitro* drug treatment protocol. Antimycin A (10 μM) was used as a positive control. The treated samples were then washed with HBSS before being incubated with 3 μM MitoSOX Red for 5 min at room temperature in the absence of light. CFs were recorded for 5 min using WinFluor V4 0.8 live cell imaging software. Signals were normalized by selecting 10 cells from each sample and measuring fluorescence intensity for each cell. A blinded process was used for cell selection. Fluorescence intensity was then measured using ImageJ software (25).

### Statistics

Data are presented as mean values ± S.E.M of n observations, where n represents the number of samples. Comparisons were assessed by one-way ANOVA with *post-hoc* Dunnett's-test using GraphPad Prism (version 7.0a). *P*-values < 0.05 were considered significant.

## Results

### Sunitinib Treatment Alters Cardiac Fibroblast Phenotype

**Figure 1:**
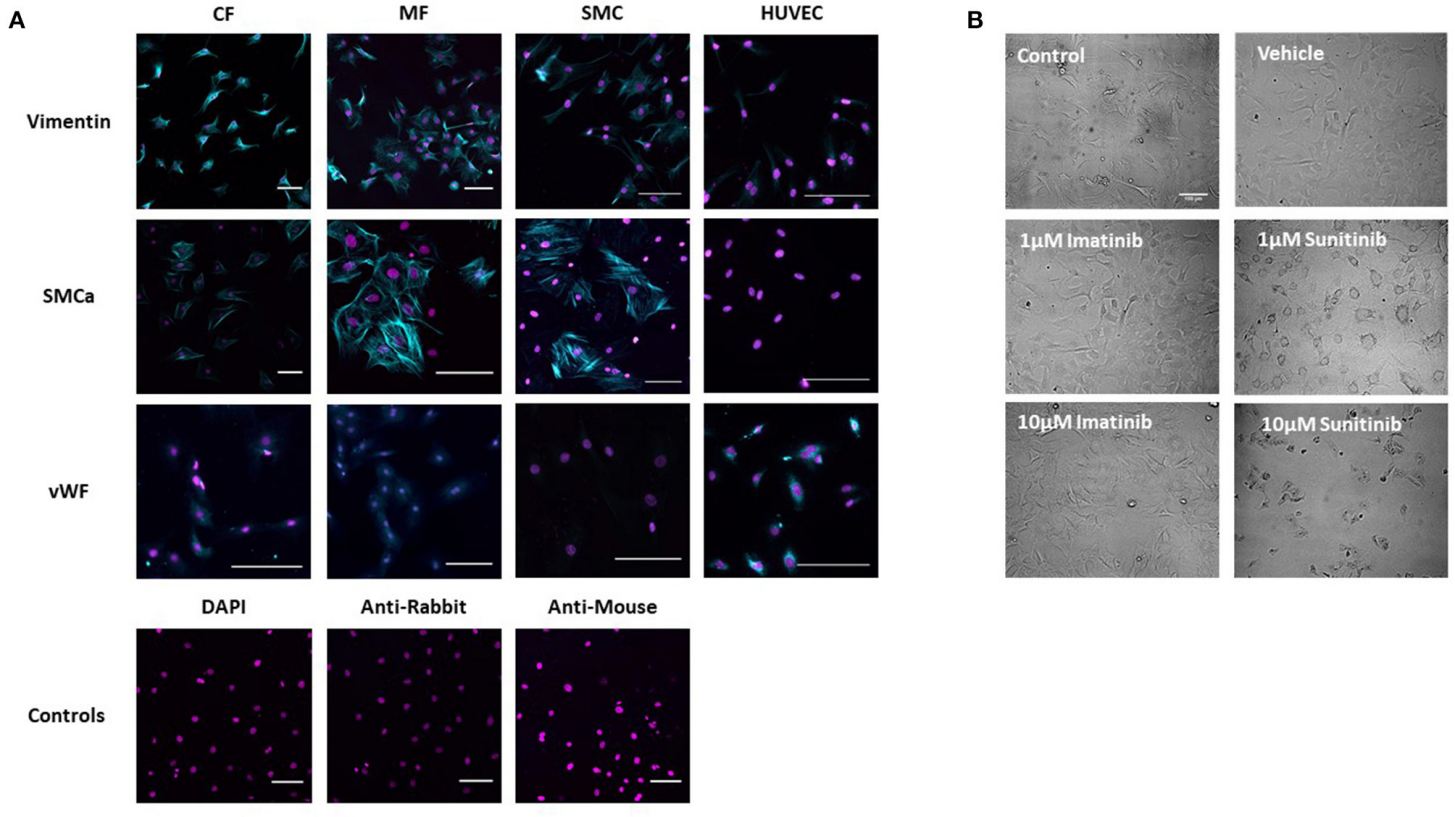
Assessment of Cardiac Fibroblast phenotype. **(A)** CF phenotype was confirmed *via* immunohistochemistry by staining for vimentin, smooth muscle cell alpha actin (SMCa) and von Willebrand Factor (vWF). Staining was compared between CFs and myofibroblasts (MF). Smooth muscle cells (SMC) and human umbilical vein endothelial cells (HUVECs) were used as positive controls. Cells treated with DAPI alone and secondary antibodies alone were used as negative controls. **(B)** Brightfield images showing CF treated with sunitinib and imatinib (0.1–10 μM) for 18 h in the presence of serum. Scale bar 100 μm. Images are from one experiment, representative of two others.

The phenotype of healthy CFs was first established *via* immunohistochemistry and brightfield imaging. Immunohistological staining showed a strong positive stain for vimentin in untreated isolated CFs, consistent with healthy, undifferentiated CFs. To exclude the possibility of contamination with SMCs or ECs, CFs were also stained for smooth muscle cell alpha actin (SMCa) (a marker of smooth muscle cell phenotype) and von Willebrand Factor (vWF) (a marker of endothelial cell phenotype). Staining was compared across CFs, myofibroblasts (MFs), SMCs and ECs. Although a positive stain for both SMCa and vWF was obtained in CFs, staining was significantly less than that obtained in the positive controls for SMCs and of a very different pattern than that seen in the positive controls for HUVECs. Staining was also less than that obtained in the phenotypically transformed MFs (Figure 1A).

In order to assess the effects of imatinib and sunitinib on CF phenotype, cells were treated with increasing concentrations of drug (1–10 μM) for 18 h and then subjected to brightfield imaging (Figure 1B). Sunitinib-induced changes in CF phenotype were evident at 1 μM sunitinib and became more apparent as concentrations increased. Sunitinib-treated CFs appeared larger and more transparent, with the formation of vacuole-like structures evident in the main cell body. Obvious cell loss was apparent at 10 μM sunitinib. The observed changes in CF phenotype were associated with subtle changes in cell marker expression determined *via* quantitative immunoblotting, although these changes were not statistically significant (data not shown). Imatinib treatment did not have any discernible effect on CF phenotype until much higher concentrations.

### Sunitinib Treatment Causes Cardiac Fibroblast Cell Death

**Figure 2:**
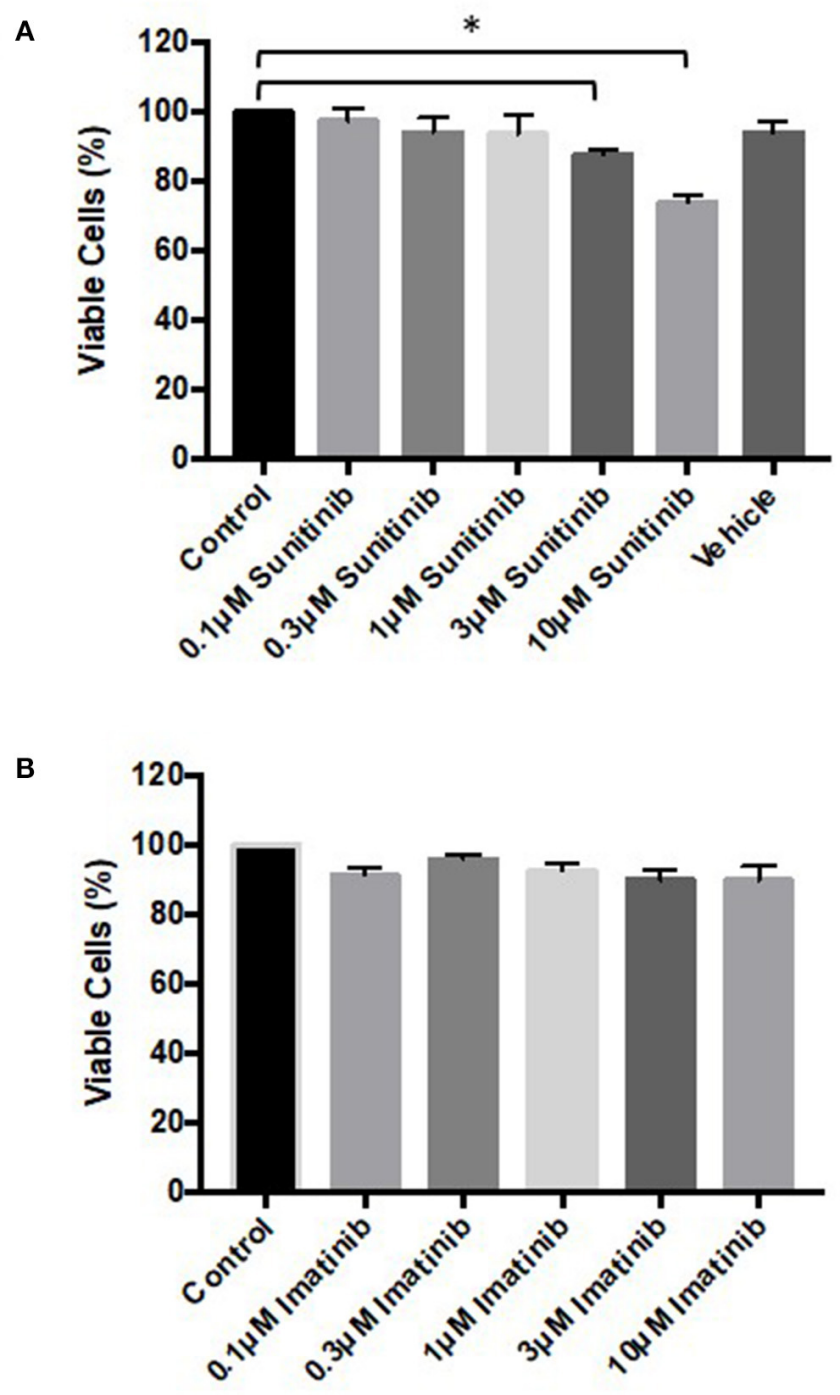
Sunitinib treatment reduces cardiac fibroblast viability. Cells were treated with sunitinib or imatinib (0.1–10 μM) in the presence of serum for 18 h. Cell proliferation was assessed *via* MTT assays. **(A)** Sunitinib treated CF **(B)** imatinib treated CF. Results are expressed as mean ± S.E.M and are normalized to control (*n* = 3, *p < 0.05).

To determine whether the changes in phenotype correlated with reduced cell viability, MTT assays and FACS analysis were performed. MTT assays revealed that sunitinib, but not imatinib, reduced CF viability (Figure 2). CF viability was significantly reduced at 3 and 10μM sunitinib, consistent with the cell loss observed in brightfield imaging.

**Figure 3:**
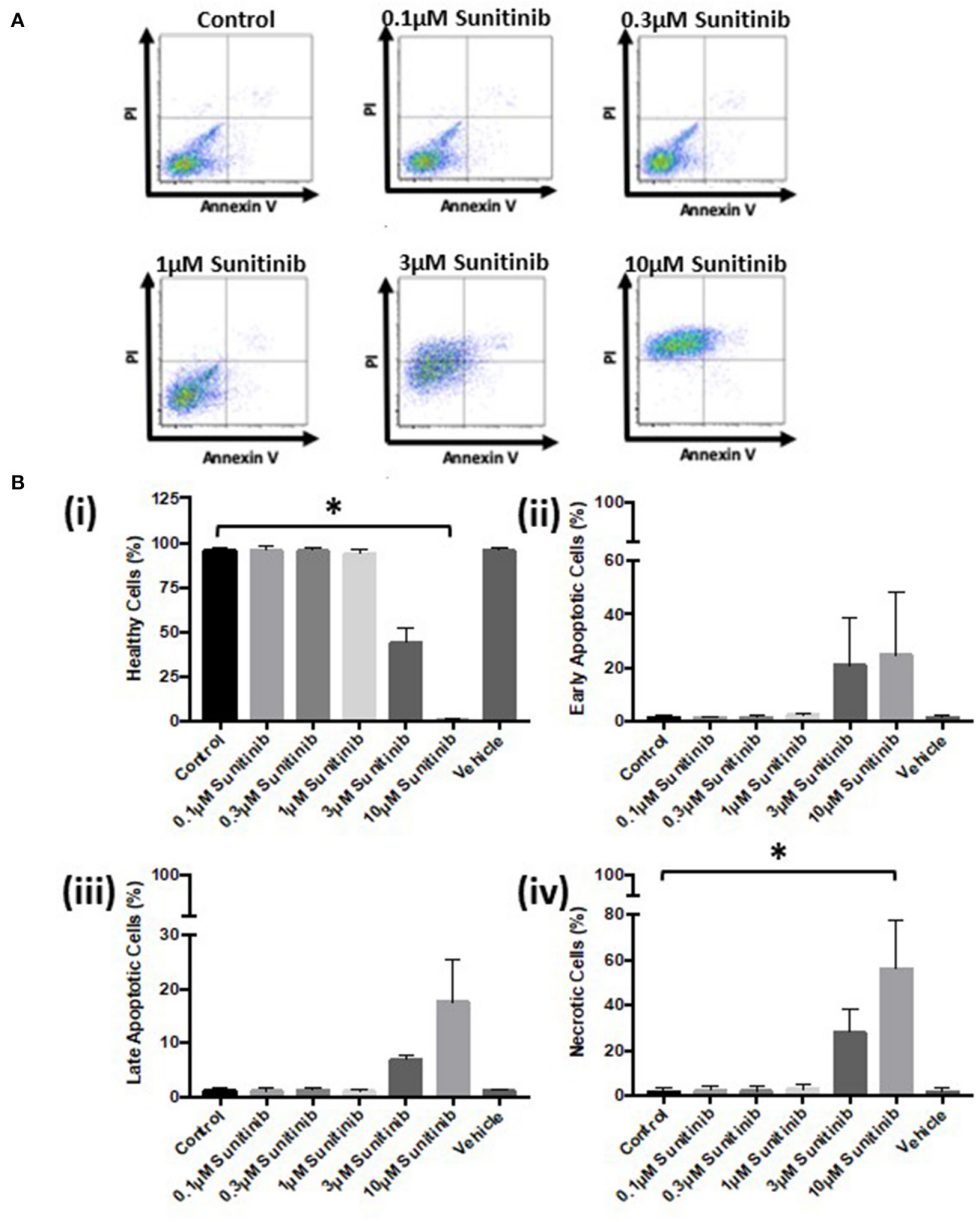
Sunitinib treatment causes increased necrosis in cardiac fibroblasts. Cells were treated with sunitinib (0.1–10 μM) in the presence of serum for 18 h. Cell viability was determined by PI and AnV staining. **(A)** Representative flow cytometry dot plots with double Annexin V-APC/PI staining for cells treated with sunitinib. **(B)** Histograms detailing the percentage of **(i)** healthy **(ii)** early apoptotic **(iii)** late apoptotic and **(iv)** necrotic cells following sunitinib treatment. Results are expressed as mean ± S.E.M (*n* = 3, *p < 0.05).

**Figure 4:**
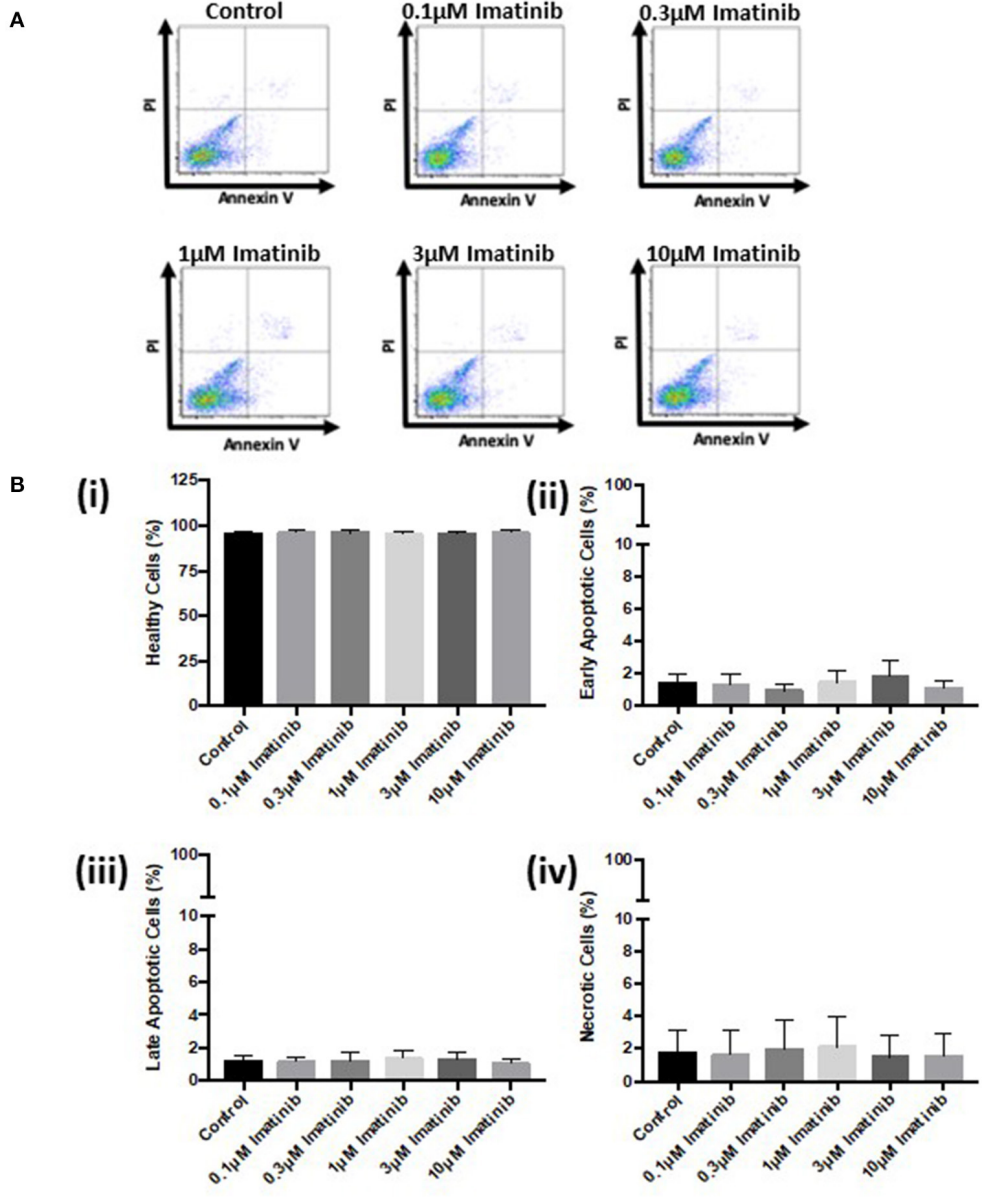
Imatinib treatment does not cause apoptosis or necrosis in cardiac fibroblasts. Cells were treated with imatinib (0.1–10 μM) in the presence of serum for 18 h. Cell viability was then determined by PI and AnV staining. **(A)** Representative flow cytometry dot plots with double Annexin V-APC/PI staining for cells treated with sunitinib. **(B)** Histograms detailing the percentage of **(i)** healthy **(ii)** early apoptotic **(iii)** late apoptotic and **(iv)** necrotic cells following imatinib treatment. Results are expressed as mean ± S.E.M (*n* = 3).

Further analysis *via* FACS confirmed these findings. Sunitinib treatment caused a reduction in the number of healthy cells (Figure 3B) with a concomitant increase in the number of early and late apoptotic cells at 3 and 10 μM treatments, as well as an increase in necrotic cells at 3μM reaching significance for necrosis at 10 μM sunitinib (Figure 3B). Imatinib treatment had no significant effect on CF viability (Figure 4).

### TKI Treatment Increases Angiotensin II-Induced Ca^2+^ Mobilization in Cardiac Fibroblasts

Altered Ca^2+^ handling is known to be associated with cell injury and death. Given the effects of TKI treatment, particularly sunitinib, on CF phenotype and viability (Figures 1–4), the possibility that either drug may affect Ca^2+^ mobilization in CFs was investigated. The effects of TKI pre-treatment on agonist-induced Ca^2+^ mobilization were examined. We have previously used Angiotensin II (AngII) to induce intracellular Ca^2+^ release in CFs (26) so this was applied to CFs in the current study to elicit responses.

**Figure 5:**
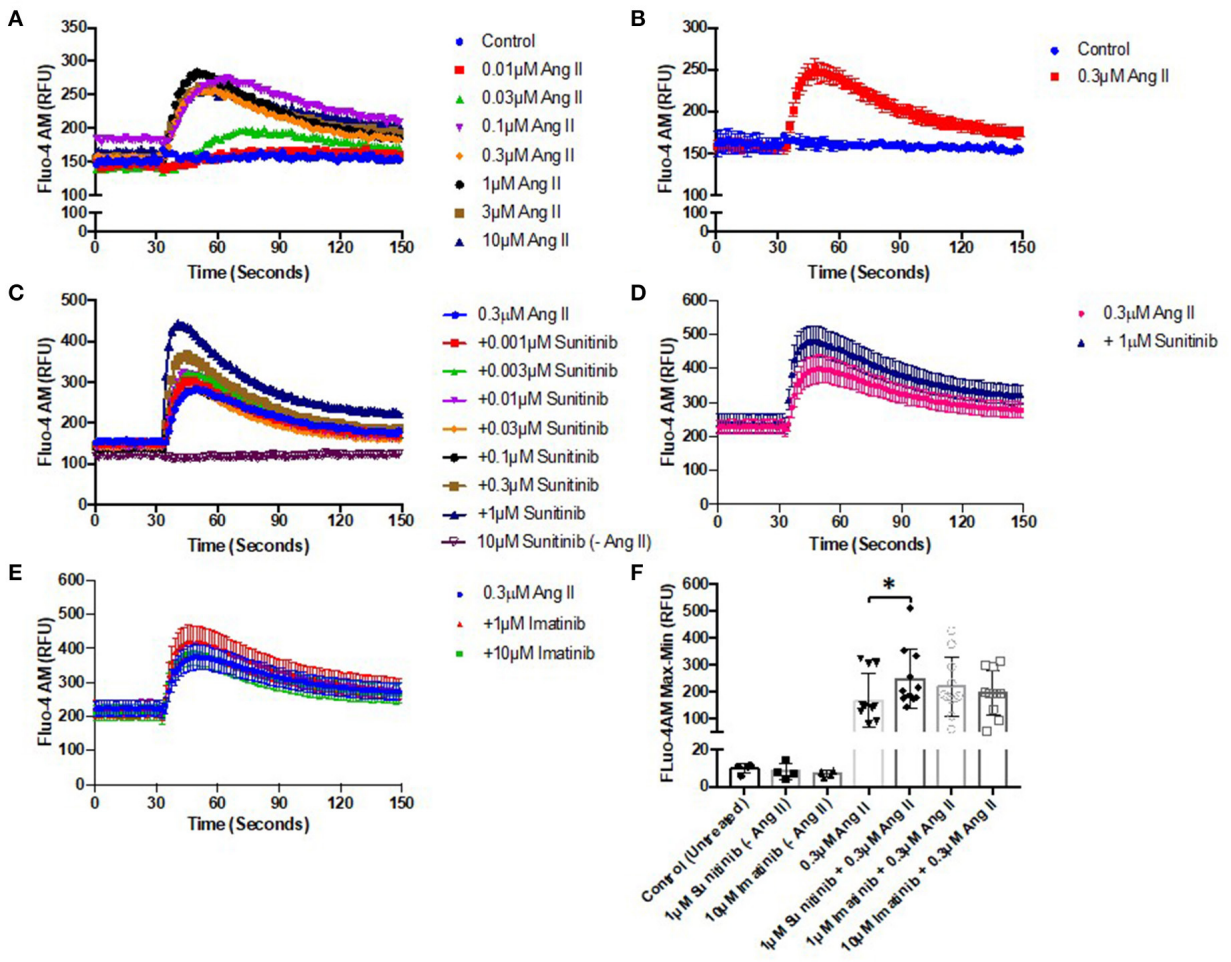
Sunitinib increases Angiotensin II—induced Ca^2+^ mobilization in cardiac fibroblasts. **(A)** Effect of increased AngII concentrations (0.01–10 μM) on intracellular Ca^2+^ mobilization **(B)** Representative trace of Ca^2+^ mobilization in response to 0.3 μM AngII stimulation **(C)** Effects of increasing concentrations of sunitinib (0.001–1 μM) on AngII induced Ca^2+^ mobilization. Sunitinib in the absence of Ang II is also shown. Results shown are mean data from one experiment, representative of five other experiments. **(D)** Comparison of maximal (1 μM) sunitinib Ang II-induced Ca^2+^ mobilization vs. control. **(E)** Effects of increasing concentrations of imatinib (1–10 μM) on AngII induced Ca^2+^ mobilization. **(F)** Effects of TKI-treatment on peak agonist-induced Ca^2+^ mobilization. Calcium mobilization was determined by measuring fluorescence at Ex:494/Em:525 nm. Results are expressed as mean ± S.E.M of 6 biological replicates, *p < 0.05.

AngII was first applied to CFs across a range of concentrations (0.01–10 μM) to determine a suitable concentration that would elicit a sub-maximal Ca^2+^ release in CFs (Figures 5A,B). Ang II at 0.3 μM elicited a rapid release of Ca^2+^ with a peak of ~80% of maximum. Using this sub-maximal concentration ensured that any subsequent effects of TKI pre-treatment (stimulatory or inhibitory) could be determined. CFs were pre-treated with a range of concentrations of either sunitinib (0.001–1 μM) or imatinib (0.01–1 μM) for 18 h and were then subjected to stimulation with 0.3 μM Ang II to induce Ca^2+^ release. Responses were compared with AngII responses in untreated cells. Cells treated with sunitinib only did not evoke intracellular calcium release (Figures 5C,F). Sunitinib pre-treatment resulted in a dose-dependent increase in AngII-evoked Ca^2+^ release (Figure 5C) with significance observed at 1 μM sunitinib (Figures 5D,F). Imatinib pre-treatment, even at higher concentrations, did not result in any significant effect on AngII-evoked Ca^2+^release (Figures 5E,F).

### Sunitinib Treatment Increases Oxidized CaMKII Expression in Cardiac Fibroblasts

**Figure 6:**
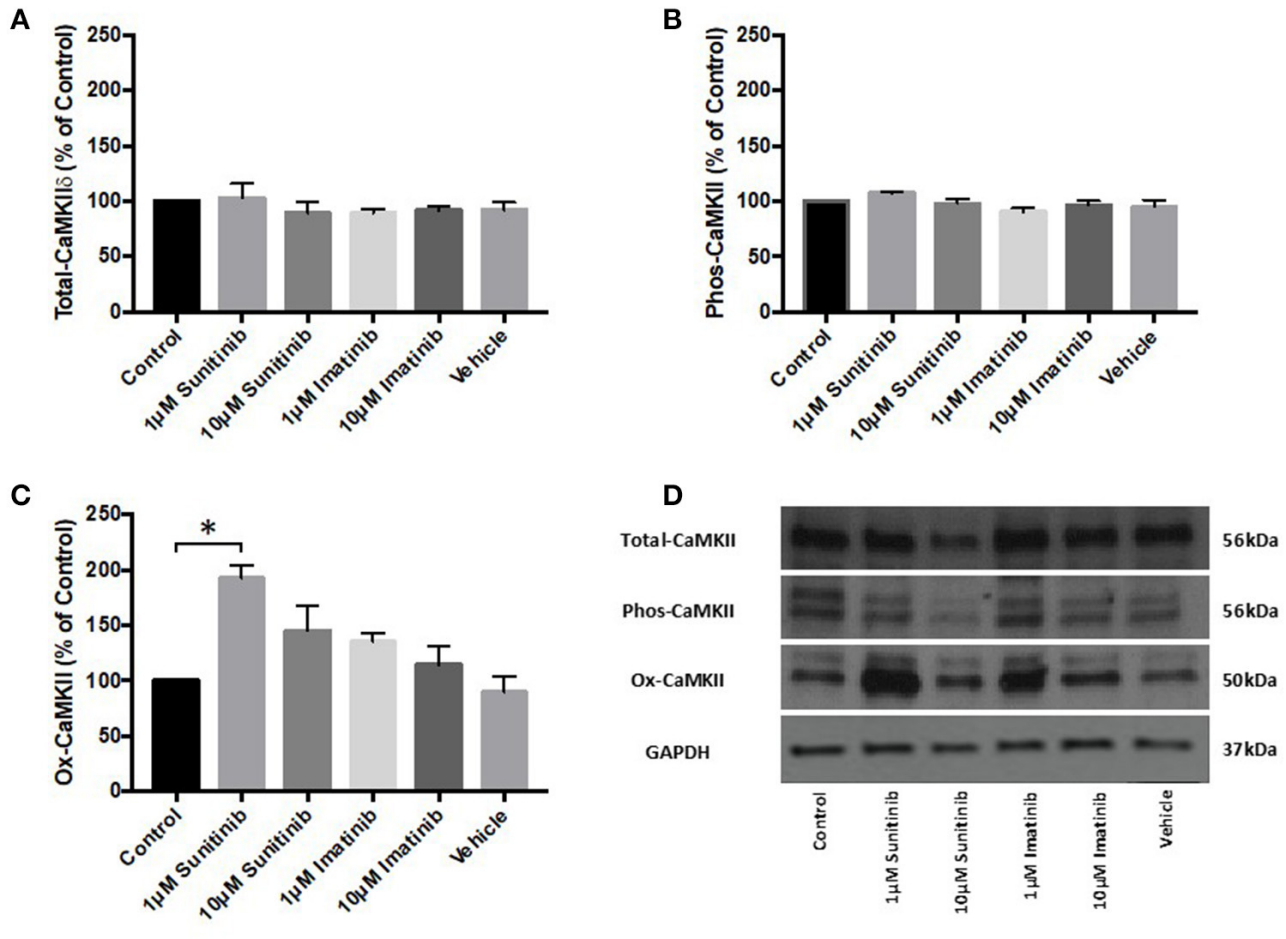
Sunitinib treatment increases ox-CaMKII expression in cardiac fibroblasts. Following the indicated treatments with either sunitinib or imatinib, CaMKIIδ expression or CaMKII activation *via* phosphorylation or oxidation was determined *via* quantitative immunoblotting **(A)** CaMKIIδ expression, normalized to GAPDH **(B)** Phos-CaMKII expression, normalized to CaMKIIδ **(C)** Ox-CaMKII expression, normalized to CaMKIIδ **(D)** Representative immunoblot showing CaMKIIδ expression and CaMKII activation following TKI treatment. Results are expressed as mean ratios protein: GAPDH ± S.E.M and are normalized to control (*n* = 3, *p < 0.05).

The effects of TKI treatment on agonist-induced intracellular Ca^2+^ mobilization, combined with the effects on cell viability, raised the possibility that TKIs may affect Ca^2+^ dependent processes within CFs and, in particular Ca^2+^-dependent protein activation. Previous work has shown that the Ca^2+^ dependent protein kinase, CaMKII, is activated and expression levels of the delta isoform (CaMKIIδ) are significantly increased in guinea pig cardiac homogenates following *in vivo* infusion of sunitinib and imatinib (22). We therefore examined the effects of chronic (18 h) treatment of CFs with both TKIs (at 1 and 10 μM) on CaMKIIδ expression and CaMKII activation (*via* either phosphorylation or oxidation). TKI treatment of CFs did not alter CaMKIIδ expression (Figure 6A) nor was phosphorylation of CaMKII affected. Interestingly though, sunitinib treatment did increase CaMKII activation *via* oxidation at 1 μM sunitinib. There was a subsequent decrease in oxidation at 10 μM sunitinib likely due to cell death as indicated by the lower signal for GAPDH (Figures 6C,D). Imatinib treatment had no discernible effect on CaMKII expression or activation.

### TKI Treatment Increases Mitochondrial Superoxide Production That Is Reversed With CaMKII Inhibition

Since sunitinib can cause activation of CaMKII *via* oxidation in CFs, we next explored whether TKI treatment may result in elevated levels of reactive oxygen species (ROS) in these cells. Furthermore, we were intrigued to explore the possibility that any TKI-mediated increase in ROS may be *via* CaMKII-mediated effects at the level of the mitochondria. To investigate the possible role of mitochondrial ROS in mediating the cardiotoxic mechanism of TKIs, the mitochondrial superoxide indicator, MitoSOX Red, was used to determine changes in mitochondrial superoxide production.

**Figure 7:**
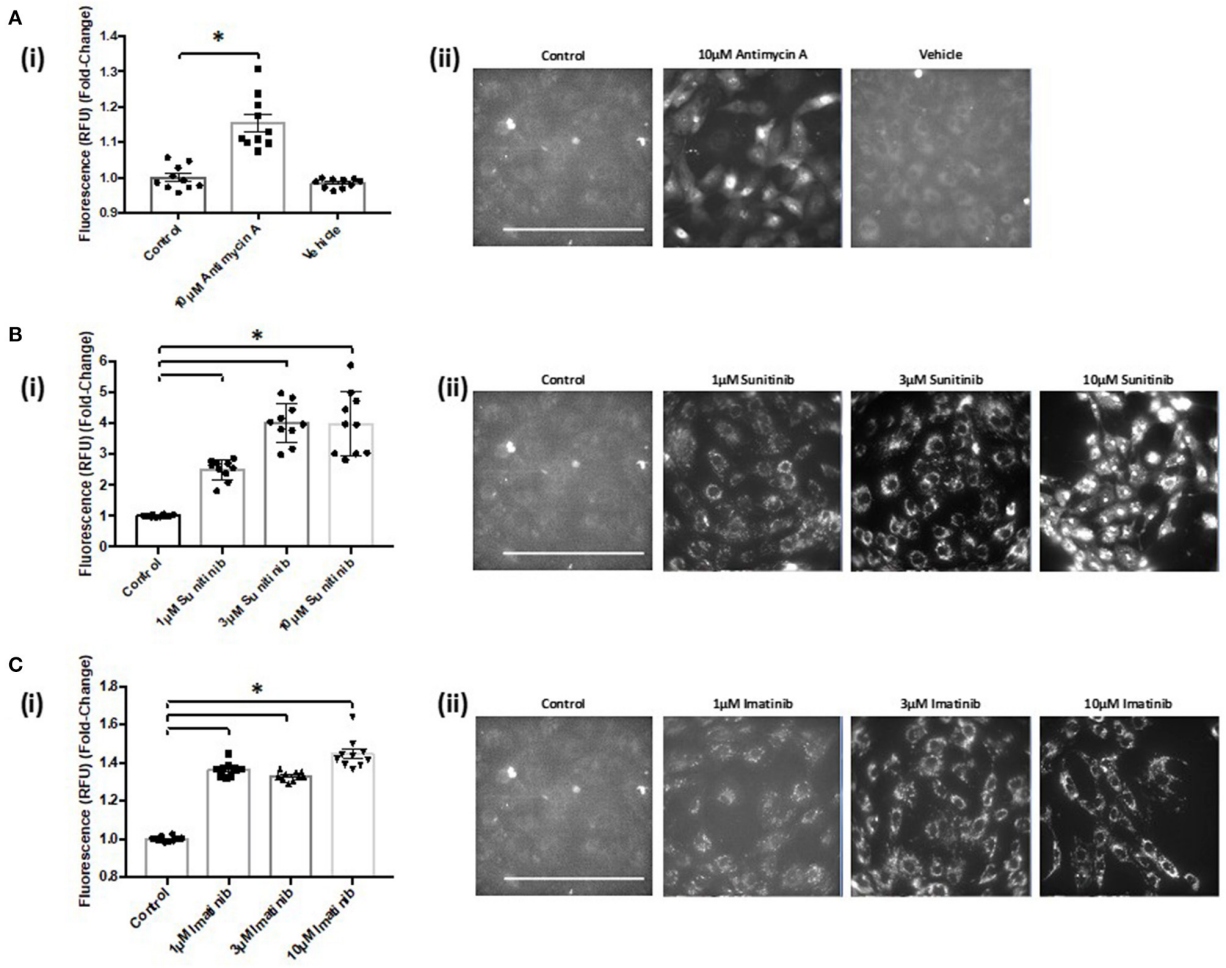
Sunitinib and imatinib treatments both cause increased mitochondrial superoxide production in cardiac fibroblasts. **(Ai)** Histogram showing superoxide production assessed via fluorescence intensity in antimycin A and vehicle treated CFs **(Aii)** Representative images of MitoSOX Red analysis of antimycin A and vehicle treated samples. **(Bi)** Superoxide production assessed via fluorescence intensity in sunitinib treated CFs **(Bii)** Representative images of MitoSOX Red analysis of sunitinib treated samples. **(Ci)** Superoxide production assessed via fluorescence intensity in imatinib treated CFs **(Cii)** Representative images of MitoSOX Red analysis of imatinib treated samples. Scale bar 100 μm. Results shown are mean data from one experiment (*n* = 10), representative of two other experiments (*p < 0.05).

**Figure 8:**
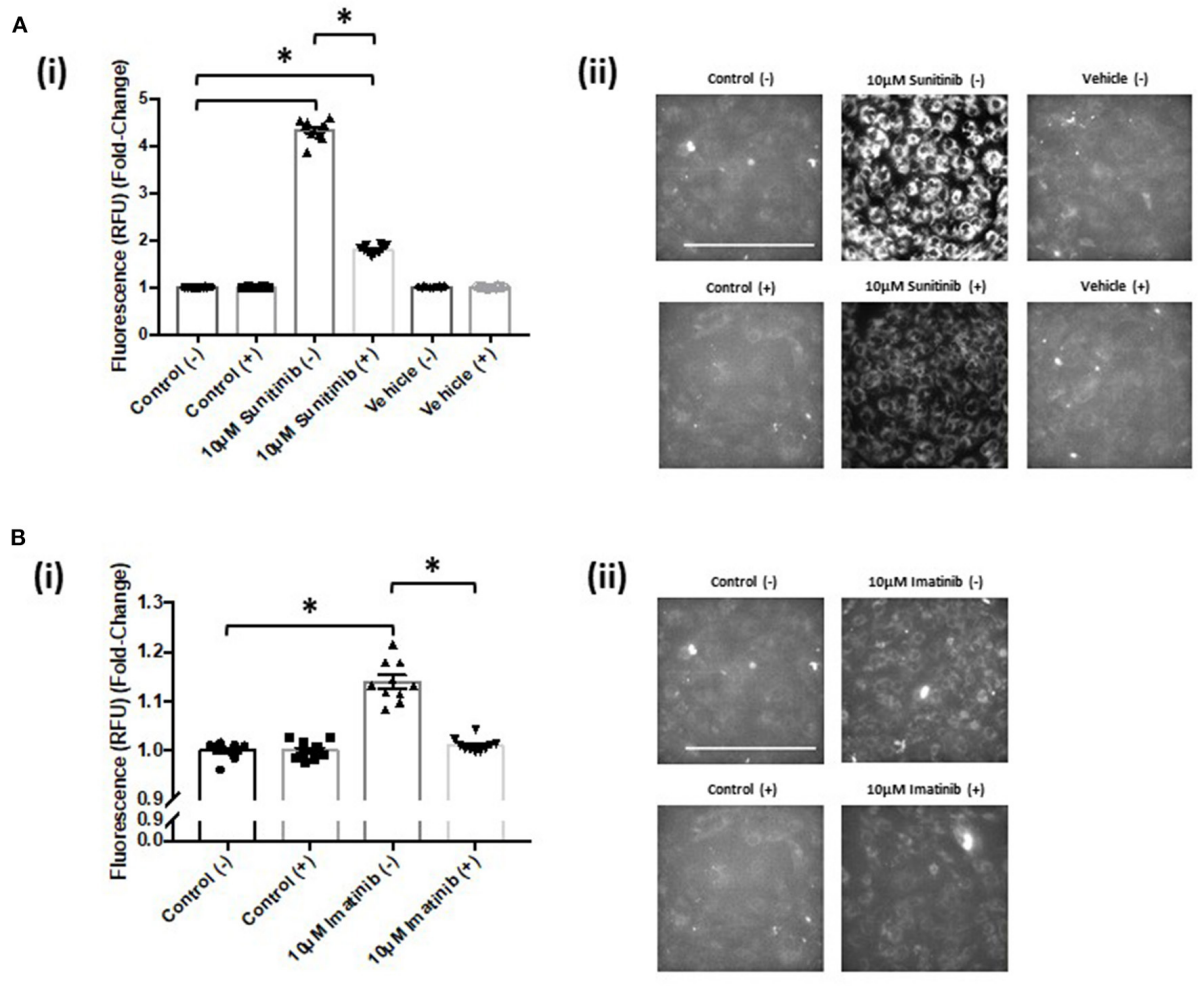
KN-93 reduces mitochondrial superoxide production in both sunitinib and imatinib treated cardiac fibroblasts. **(Ai)** Superoxide production assessed *via* fluorescence intensity in sunitinib treated CF in the presence (+) or absence (−) of 5 μM KN-93. **(Aii)** Representative images of MitoSOX Red analysis of sunitinib treated samples in the presence (+) or absence (−) of KN-93. **(Bi)** Superoxide production assessed *via* fluorescence intensity in imatinib treated CF in the presence (+) or absence (−) of KN-93. **(Bii)** Representative images of MitoSOX Red analysis of imatinib treated samples in the presence (+) or absence (−) of KN-93. Scale bar 100 μm. Results shown are mean data and images from one experiment where the same control (−) presented previously for non-KN 93 treatment has been used as a reference comparison for control (+) (*n* = 10). Imatinib and sunitinib treatments from the same experiment have been presented separately for clarity and are representative of two other experiments (*p < 0.05).

Live cell imaging revealed that both sunitinib and imatinib significantly increased mitochondrial superoxide production in CFs. This is evident at all concentrations tested and the fold-changes observed following TKI treatment were markedly higher than that observed with the mitochondrial complex III inhibitor Antimycin A, which was used as a positive control for increased superoxide production (Figures 7A(i),(ii)). Sunitinib resulted in considerably higher fluorescence than imatinib (Figures 7B(i) vs. 7C(i)), again suggesting the increased potency and cardiotoxic potential of sunitinib when compared with imatinib. Interestingly, when CFs were pre-treated with the CaMKII inhibitor KN-93, sunitinib-mediated superoxide production was significantly reduced (Figure 8A). Moreover, in cells treated with 10 μM imatinib, KN-93 pre-treatment completely abolished imatinib-induced mitochondrial superoxide production (Figure 8B).

## Discussion

In the current study we have evaluated the effects of sunitinib and imatinib on both phenotype and function of adult rat CFs to gain insight into possible mechanisms underlying the cardiotoxic profiles of both drugs in the non-contractile fibroblasts of the heart. Effects on cell phenotype, viability, intracellular Ca^2+^ release and mitochondrial superoxide production were assessed and, for the first time, a potential role for CaMKII activation *via* oxidation as an underlying mechanism of action of sunitinib in CFs was highlighted. Interestingly, but unsurprisingly, sunitinib and imatinib exerted differential effects on CFs and this may provide an indication of the broader cardiotoxic potential of each drug.

Initial characterization of CFs was performed using immunocytochemistry and this revealed distinct vimentin staining of CFs with low levels of SMCa and altered distribution of vWF confirming results from previous studies (27, 28). Importantly, the distinction between CFs and MFs was highlighted with a clear difference in phenotype and SMCa staining across the two (Figure 1A). Treatment of CFs with sunitinib and imatinib at relatively low concentrations (1–10 μM) and in the presence of serum enabled a realistic assessment of the potential for these drugs to affect CF phenotype and performance in the clinical setting. Previous investigations have often used much higher concentrations of drugs which may not be clinically relevant (29) and very few studies have assessed the effects on CFs (11). Sunitinib induced clear alterations in cell phenotype and viability at concentrations as low as 1 μM whereas imatinib effects were less pronounced and phenotypic changes only started to become evident at a 10-fold higher concentration. This pattern was maintained when the effects of both drugs on cell viability were studied in more detail. Using MTT assays and FACS analysis, a sunitinib-induced reduction in viability was observed at 3 and 10 μM with significant levels of necrosis occurring at 10 μM (Figures 2, 3). There were no significant effects of imatinib at any of the concentrations tested (Figures 2, 4). These observations fit with the differences observed in cardiotoxicity reported for these drugs in the clinic (29–31). Differences in % viability were detected across MTT and flow cytometry and this reflects the differences in sensitivity between both assays. Importantly, imatinib did not induce any measurable change in viability assessed via either assay.

Previous work by our group has highlighted that acute and chronic treatment of guinea pigs with sunitinib and imatinib can alter the expression and/or activity of CaMKII measured in ventricular cardiac homogenates (22). CaMKII is well-established as a pivotal molecule in cardiac physiology regulating cellular processes such as excitation-contraction coupling, cellular mechanics and energetics (32, 33). CaMKII is also recognized as an important mediator of pathological signaling and remodeling in disease (34, 35). As well as a recognized role in cardiomyocyte physiology and pathophysiology, a role for CaMKII in CF dysfunction has also been highlighted (27). The possibility that sunitinib and/or imatinib might alter CaMKII activity in CFs has not previously been explored but this could be a central factor contributing to the underlying mechanism of cardiotoxicity. In particular, given the contribution that CFs have to extracellular matrix deposition and to cardiac myocyte function, detrimental effects on CF viability by TKIs could impact directly upon cardiac contractility leading in particular to impaired diastolic function. Interestingly, sunitinib was able to increase AngII-mediated intracellular Ca^2+^ release and this trend was evident at all concentrations of sunitinib tested (0.001–1 μM), becoming significant at 1 μM. Lower concentrations were used in these experiments to closely mirror plasma concentrations that are seen in patients (24, 36) and to investigate whether intracellular signaling (in the form of Ca^2+^ release) may be more sensitive to drug-induced effects than those effects observed phenotypically and functionally. Although imatinib appeared to increase intracellular Ca^2+^, this effect was not significant even up to 10 μM imatinib. This raises the potential that sunitinib (and possibly imatinib) may have for exacerbating Ca^2+^-activated responses. The contribution that excess Ca^2+^ release has toward apoptosis and necrosis is well-documented (37, 38). Sustained CaMKII activation is a central feature of these pathological processes and the enzyme is initially activated in response to increased intracellular [Ca^2+^]. CaMKII can also be activated by post-translational modifications downstream of Ca^2+^/calmodulin binding. This can include autophosphorylation, oxidation, *S*-nitrosylation and *O*-GlcNAcylation. All of these modifications can occur during sustained and pathological activation (39). Here, for the first time, we have investigated the ability of both sunitinib and imatinib to induce either phosphorylation or oxidation of CaMKII. As well as the possibility that both drugs may activate CaMKII *via* elevations in intracellular Ca^2+^, data shown here suggest that sunitinib can also cause oxidation of CaMKII in CFs (Figure 6). Oxidation of CaMKII is able to alter the Ca^2+^ sensitivity of the enzyme enabling activation at low intracellular [Ca^2+^] and sustaining activity in the absence of Ca^2+^/calmodulin (40, 41) and this may be a means by which the cardiotoxic effects of CaMKII are harnessed by certain anti-cancer drugs. This mode of activation of CaMKII may be a distinguishing feature in differences in mechanism of action between the two TKIs although this would require further investigation. Interestingly, CaMKII has been linked to activation of a regulated form of necrosis, termed necroptosis (33). Necroptosis can be triggered *via* activation of receptor-interacting protein (RIP3) which has CaMKII as a substrate, and it has been shown that disruption of either RIP3 or CaMKII can significantly reduce cell death (42). Here we have shown that sunitinib can induce necrosis (we have not assessed necroptosis) and can activate CaMKII. It is possible that the two processes are linked in the mechanism of action of sunitinib-induced cardiotoxicity in CFs.

**Figure 9:**
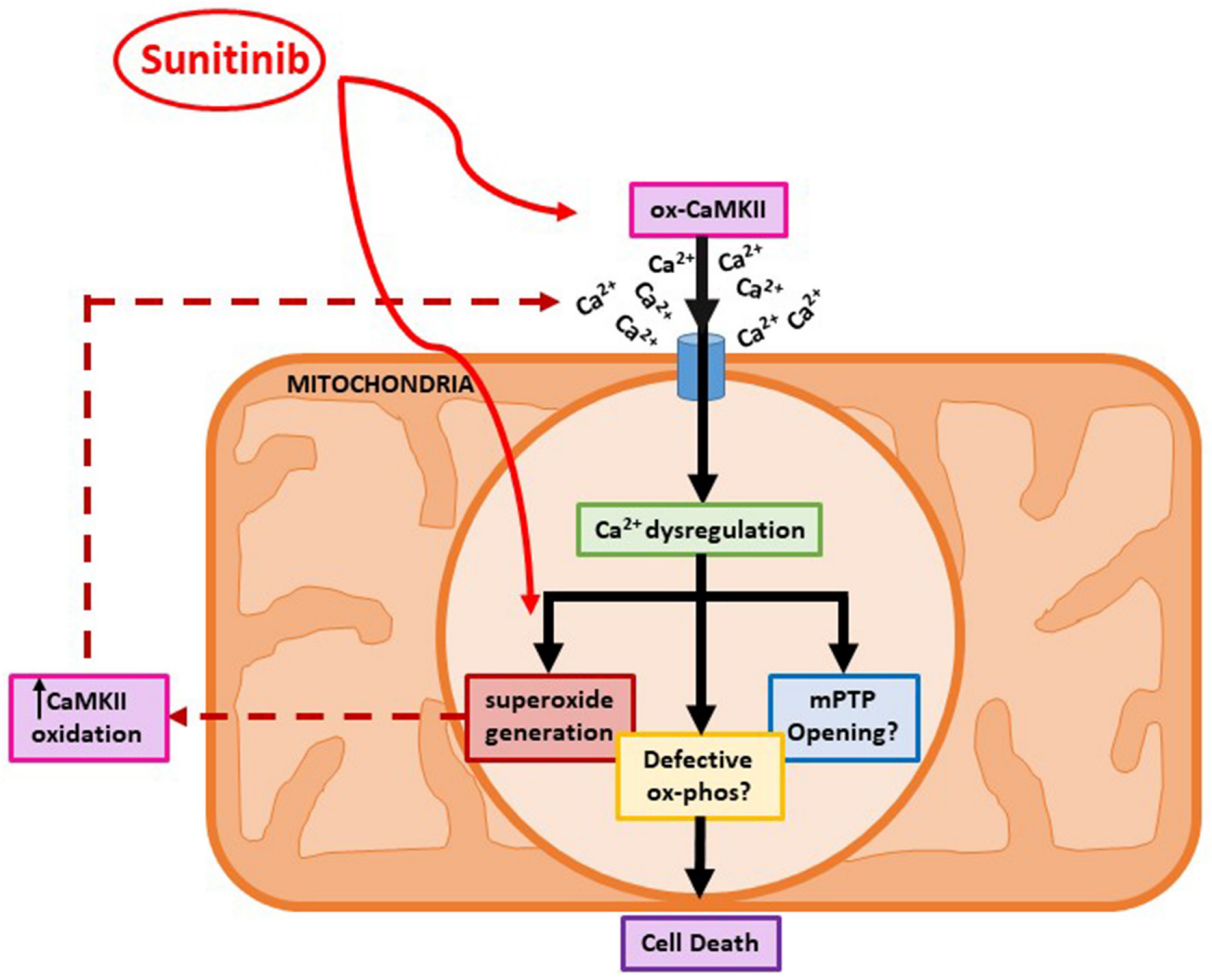
A proposed mechanism for sunitinib-induced mitochondrial dysfunction in adult cardiac fibroblasts. Schematic showing proposed sunitinib-induced events at the level of the mitochondria in CFs. Sunitinib treatment may exert direct effects on mitochondrial superoxide production that can then result in CaMKII oxidation. Sunitinib-induced increases in CaMKII oxidation and activation can then go on to cause elevated superoxide production so a positive feedback loop may exist exacerbating the cardiotoxic effects. The combination of elevated CaMKII oxidation and increased superoxide production ultimately causes toxicity at the level of the mitochondria and can contribute to CF cell death.

Since increased oxidation of CaMKII was evident following sunitinib treatment, we investigated whether either sunitinib or imatinib treatment could result in increased levels of mitochondrial superoxide. Although the role of ROS in cardiac pathophysiology remains unclear, ROS have been implicated in a variety of processes that affect cardiac function, both contractile and non-contractile processes (43, 44). While ROS have been implicated in the pathogenesis of anthracycline induced cardiotoxicity, there is less evidence of the effects of ROS in TKI-induced cardiotoxicity, particularly relating to effects on non-contractile cells of the heart. Importantly, current evidence suggests that anti-oxidant therapy does not offer cardioprotection against anthracycline-induced cardiotoxicity (5). Therefore, a better understanding of how ROS production may be mediated by anti-cancer agents and relate to cardiotoxic effects is required. Here we have shown that both sunitinib and imatinib can induce significant increases in mitochondrial superoxide in CFs following 18 h treatment and this occurs across all concentrations tested (1–10 μM) (Figure 7). Interestingly, when we pre-treat CFs with a CaMKII inhibitor (KN-93) and repeat TKI treatment at the highest concentrations of TKI tested (10 μM), the effects of both sunitinib and imatinib on mitochondrial superoxide production are abrogated (Figure 8). It is likely that KN-93 will inhibit global CaMKII in the CF and this may explain why we see an effect on imatinib induced superoxide production in the absence of any quantifiable CaMKII activation in this study. These results highlight that interventions interfering with CaMKII targeting to the mitochondria or with CaMKII oxidation are worthy of further study. Methionine sulfoxide reductase A (MsrA) can reduce oxidized CaMKII and could be a promising target to limit TKI-induced oxidative stress (40). It is possible that sunitinib-induced effects on CaMKII oxidation and superoxide production are not mutually exclusive and one may exacerbate the other driving toxicity (Figure 9). These mitochondrial events are already known to be a central feature of myocardial disease (45).

Recent work using transgenic mice and computational modeling has shown that myocardial infarction surgery causes significant elevation of mitochondrial CaMKII along with left ventricular dilatation. Pathological remodeling is reversed in mice with genetic mitochondrial CaMKII inhibition (46). The significance of mitochondrial targeted CaMKII activation and inhibition in the switch between normal function and pathological consequences is striking and is pertinent to the results presented in the current study.

## Conclusion

For the first time we have demonstrated differential cardiotoxic effects in adult CFs in response to sunitinib and imatinib at low, clinically relevant concentrations of each drug. We have shown that sunitinib is capable of mediating an increase in agonist-induced [Ca^2+^]_i_ and can activate CaMKII *via* oxidation. Both drugs lead to significant elevation of mitochondrial superoxide production and this is reversed in the presence of CaMKII inhibition. The implications of these results suggest that TKI-mediated cardiotoxicity could be reduced or reversed *via* targeted CaMKII inhibition. Development of CaMKII inhibitors to target heart disease is already ongoing. The use of more targeted CaMKII inhibitors in the treatment of anti-cancer drug cardiotoxicity could be an additional niche area for the pharmaceutical industry and fundamentally, could represent a new and important therapeutic approach in the field of cardio-oncology.

## Data Availability Statement

The raw data supporting the conclusions of this article will be made available by the authors, without undue reservation.

## Author Contributions

SCu and MC worked on the conception and organization of the research project. Experimental work was conducted predominantly by CM. Data analysis was conducted by CM, SCh, RW, and MC. SCu and CM wrote the first draft of the manuscript. MC and SCh reviewed the manuscript. All authors read and approved the final version of the manuscript.

## Conflict of Interest

The authors declare that the research was conducted in the absence of any commercial or financial relationships that could be construed as a potential conflict of interest.
